# The Role of Religiosity in Anxiety and Life Satisfaction: Insights from Sociodemopgraphic Data

**DOI:** 10.1192/j.eurpsy.2025.405

**Published:** 2025-08-26

**Authors:** E. D. D. Cindik-Herbrueggen

**Affiliations:** Psychiatry, Neuro-Psychiatric Center, München, Germany

## Abstract

**Introduction:**

The connection between well-being and various sociopsychological factors such as age, gender, education level, and more is a growing and significant area of interest in today’s studies.

**Objectives:**

The aim of this study is to examine the relationship between sociodemographic factors, life satisfaction, levels of anxiety, and religiosity. To achieve this goal, the study explored whether religiosity has an effect on anxiety and life satisfaction on the one hand, and whether sociodemographic variables influence the centrality of religion in participants’ lives on the other.

**Methods:**

To ensure honest responses, the surveys were filled out anonymously. Statistical analyses were conducted using the SPSS program, applying Pearson Correlation and Analysis of Variance (ANOVA) to determine if there was a correlation between sociodemographic data and questionnaire scores.

**Results:**

In the Analysis of Variance (ANOVA) of total STAI-G scores and religiosity, a significance level of 0.010 was observed, while the Post-Hoc Test suggested that this significance may be found between atheist participants and those who identified with a religion other than Islam, Christianity, or Judaism, with a value of 0.019. Additionally, there is a strong significant correlation of 0.018 between participants’ native language and total CRS-10 scores, which may imply that native language, encompassing important factors like cultural background, can influence participants’ religious beliefs and practices. Lastly, a significant correlation of 0.041 was found between alcohol consumption and total CRS-10 scores. This correlation could indicate that religious participants are more likely to consume less alcohol compared to non-religious participants, possibly due to their religious beliefs.

**Conclusions:**

This study highlights the importance of examining the connections between sociodemographic factors, life satisfaction, anxiety, and religiosity in relation to mental well-being. The findings aim to provide useful guidance for the evolution of interventions that focus on and enhance well-being and continuity, while emphasizing the quality of life experienced by patients from diverse cultures and backgrounds.

**Disclosure of Interest:**

None Declared

